# Supplementary material for: A Highly Effective Bacteriophage-1252 to Control Multiple Serovars of Salmonella enterica
Source: Foods. 2023 Feb 13;12(4):797. doi: 10.3390/foods12040797 (PMC9955900; doi:10.3390/foods12040797)
Supplement: Supplementary file 1 [file foods-12-00797-s001.zip › foods-2097979-supplementary.pdf]

Table S1 Sources of samples and bacterial host cell analysis.

Sources	Sample No.	*Salmonella* spp. (bacterial host cell)
Pond water (Farm #1)	1208	-
	1209	-
	1210	-
	1211	-
	1212	+
	1213	+
	1214	-
	1215	-
	1216	+
	1217	+
	1218	-
	1219	-
Cattle feces (Farm #1)	1220	+
	1221	+
	1222	-
	1223	+
	1224	-
	1225	+
	1226	-
	1227	-
	1228	-
	1229	-
Cattle drinking water (Farm #2)	1240	+
	1241	-
	1242	+
	1243	-
	1244	-
	1245	-
	1246	-
	1247	+
	1248	-
	1249	+
Cattle lagoon water (Farm #2)	1250	-
	1251	-
	1252	+
	1253	+
	1254	+
	1255	+
	1256	+
	1257	+
	1258	+
	1259	+
Cattle feces (Farm #2)	1335	+
	1336	+

	1337	+
	1338	+
	1339	+
Chicken feces (Farm #3)	CF	+
Chicken water (Farm #3)	CW	+
Turkey feces (Farm #4)	2902	+

"+", *Salmonella*-specific phage was identified and isolated from farm the samples; "-", no phages were identified from the farm sample.
